# Anticoagulation control among patients on vitamin K antagonists in nine countries in Sub-Saharan Africa

**DOI:** 10.1007/s11239-023-02928-1

**Published:** 2024-03-13

**Authors:** Julius Chacha Mwita, Joel Msafiri Francis, Chriselda Pillay, Okechukwu S. Ogah, Dejuma Yadeta Goshu, Francis Agyekum, John Mukuka Musonda, Maduka Chiedozie James, Endale Tefera, Tsie Kabo, Keolebile Irene Ditlhabolo, Kagiso Ndlovu, Ayoola Yekeen Ayodele, Wigilya P. Mikomangwa, Pilly Chillo, Albertino Damasceno, Aba Ankomaba Folson, Anthony Oyekunle, Erius Tebuka, Fredrick Kalokola, Karen Forrest, Helena Dunn, Kamilu Karaye, Fina Lubaki Jean-Pierre, Chala Fekadu Oljira, Tamrat Assefa, Tolulope Shogade Taiwo, Chibuike E. Nwafor, Olufemi Omole, Raphael Anakwue, Karen Cohen

**Affiliations:** 1 Department of Internal Medicine, University of Botswana and Princess Marina Hospital, Gaborone, Botswana; 2 Department of Family Medicine and Primary Care School of Clinical Medicine, Faculty of Health Sciences, University of the Witwatersrand, Johannesburg, South Africa; 3 Division of Clinical Pharmacology, Department of Medicine, University of Cape Town, Cape Town, South Africa; 4 Cardiology Unit, Department of Medicine, University of Ibadan/University College Hospital, Ibadan, Oyo State Nigeria; 5 Department of Internal Medicine, College of Health Sciences, Addis Ababa University, Addis Ababa, Ethiopia; 6 Department of Medicine, College of Health Sciences, Korlebu Teaching Hospital, University of Ghana, Ghana, Ethiopia; 7 Federal Medical Centre (FMC), Umuahia, Abia State Nigeria; 8 Department of Paediatrics and Adolescent Health, University of Botswana and Princess Marina Hospital, Gaborone, Botswana; 9 Department of Computer Science, University of Botswana, Gaborone, Botswana; 10 Cardiology Unit, Department of Medicine, Federal Teaching Hospital, Gombe, Gombe State Nigeria; 11 Department of Clinical Pharmacy and Pharmacology, Muhimbili University of Health and Allied Sciences, Dar es Salaam, Tanzania; 12 Department of Internal Medicine, Muhimbili University of Health and Allied Science, Dar es Salaam, Tanzania; 13 Faculty of Medicine, University Eduardo Mondlane, Maputo, Mozambique; 14 University of Health and Allied Sciences, Accra, Ghana; 15 Department of Internal Medicine, Bugando Medical Centre, Mwanza, Tanzania; 16 MRC Unit The Gambia at LSHTM, Fajara, The Gambia; 17 Department of Medicine, Bayero University & Aminu Kano Teaching Hospital, Kano, Nigeria; 18 Department of Family Medicine and Primary Care, The Protestant University of Congo, Kinshasa, Democratic Republic of the Congo; 19 Department of Pharmacology and Clinical Pharmacy, College of Health Sciences, Addis Ababa University, Addis Ababa, Ethiopia; 20 University of Uyo Teaching Hospital, Uyo, Nigeria; 21 The University of Port Harcourt Teaching Hospital, Port Harcourt, Nigeria; 22 Departments of Medicine, Pharmacology/Therapeutics, The University of Nigeria Teaching Hospital, Enugu, Nigeria

**Keywords:** Vitamin K antagonists, VKA, Anticoagulation control, Sub-Saharan Africa

## Abstract

Vitamin K antagonists (VKA) is the primary anticoagulant in most settings of Sub-Saharan Africa. Understanding the quality of anticoagulation services in the continent is vital in optimising the intended benefits. This study assessed the quality of anticoagulation and associated factors among VKA-treated patients in nine SSA countries. We conducted a retrospective cohort study of randomly selected patients on anticoagulation from 20 clinics in Botswana, the Democratic Republic of Congo, Ethiopia, Gambia, Ghana, Mozambique, Nigeria, Tanzania, and South Africa. Eligible participants were those on VKAs for at least three months and with at least four international normalised ratios (INR) results in 2019–2021. We report the proportion of INR values in the therapeutic range, time-in-therapeutic range (TTR) using the Rosendaal method, and the proportion of patients with TTR ≥ 65% (optimal anticoagulation). The mean age was 51.1(16.1) years, and 64.2% were women. The most common indications for VKA included venous thromboembolism (29.6%), prosthetic valves (26.7%) and atrial fibrillation/flutter (30.1%). We analysed 6743 INR tests from 1011 participants, and of these, 48.5% were sub-therapeutic, 34.1% therapeutic, and 17.4% were supratherapeutic relative to disease-specific reference ranges. TTR was calculated for 660 patients using 4927 INR measurements. The median (interquartile range [IQR]) TTR was 35.8(15.9,57.2) %. Optimal anticoagulation control was evident in 19.2% of participants, varying from 2.7% in Tanzania to 23.1% in Ethiopia. The proportion of patients with TTR ≥ 65% was 15,4% for prosthetic heart valves, 21.1% for venous thromboembolism and 23.7% for atrial fibrillation or flutter. Countries with universal health coverage had higher odds of optimal anticoagulation control (adjusted odds ratio (aOR) 1.79, 95% confidence interval [CI], 1.15– 2.81, p = 0.01). Patients on VKAs for different therapeutic indications in SSA had suboptimal TTR. Universal health coverage increased the odds of achieving TTR by 79%. The evidence calls for more intensive warfarin management strategies in SSA, including providing VKA services without out-of-pocket payments.

## Highlights

- The study of nine countries in SSA provided a snapshot of the quality of VKA anticoagulation in daily clinical practice for a region with similar health system challenges.
- The analysis noted a suboptimal quality of anticoagulation for different therapeutic VKA indications across selected countries.
- The median TTR was 35.8.% (recommended minimum 65%), and only 80.8% of participants had a suboptimal quality of anticoagulation.
- Countries with universal health coverage had higher odds of optimal anticoagulation control.

## Introduction

Vitamin K anticoagulants (VKAs) have been widely used for decades for treating and preventing thromboembolic complications of venous thromboembolism (VTE), atrial fibrillation (AF), and valvular heart disease [1]. Furthermore, VKAs are the only recommended anticoagulant treatment for patients with rheumatic heart disease-associated atrial fibrillation and mechanical heart valves [2]. However, VKA use can be challenging given the narrow therapeutic window, unpredictable response, and multiple interactions with other drugs and diets [3, 4]. Therefore, to attain the maximal therapeutic benefits of VKA while minimising bleeding complications, warfarin therapy must be tightly controlled and maintained within a narrow therapeutic range based on INR [5]. The recommended INR targets are 2.0–3.0 for VTE and AF and 2.5–3.5 for mechanical prosthetic valves [6, 7]. The safety and efficacy of warfarin depend on the extent to which anticoagulant regimens maintain patients in these therapeutic ranges. The average percentage of the time in the therapeutic range (TTR) and the percentage of INR values in the therapeutic range are ways to assess the quality of anticoagulation related to thrombotic and bleeding complications [5, 8]. Guideline-recommended thresholds for optimal anticoagulation are TTR ≥ 65% for the British National Institute for Health and Care Excellence and TTR ≥ 70% for the European Society of Cardiology [9, 10]. Achieving these thresholds has been challenging in most settings, leaving a substantial proportion of warfarin-treated patients sub-optimally anticoagulated and with an increased stroke, bleeding, and mortality risk [11–15]. In the Global Anticoagulant Registry in the FIELD (GARFIELD) study, the proportion of patients with TTR ≥ 65% was low, varying from 16.7% in Asia to 49.4% in Europe [13]. The situation is worse in SSA, with the proportion of patients with optimal anticoagulation control as low as 15%. [16] The long travel distance to access the few unreliable centralised VKA services, leading to the low frequency of INR monitoring, out-of-pocket expenses for VKA services and other health system-related problems that affect the accessibility of VKA services, significantly impact anticoagulation control in these settings [13, 16–18]. The resulting suboptimal quality of anticoagulation is a significant concern in SSA, given the high burden of rheumatic heart disease-associated AF and mechanical heart valve transplantation, for which VKA is the preferred oral anticoagulation strategy. Even for therapeutic indications that non-vitamin K oral anticoagulants (NOACs) are possible substitutes, VKAs remain the primary anticoagulant as the uptake of NOACs is limited by the high cost and accessibility in African settings. Consequently, VKAs could remain the primary anticoagulant in SSA for decades and efforts to achieve optimal VKA anticoagulation are crucial [13]. Evidence suggests that VKA at stable optimal anticoagulation has comparable efficacy and safety to NOACs [19–21]. The current study aimed to describe the quality of anticoagulation control among patients using VKA in nine SSA countries.

## Methods

### Study design, setting and patients

This retrospective cohort study extracted data from adults 18 years or older at eight outpatient clinics from Nigeria, four from South Africa, two from Tanzania and one each from Botswana, the Democratic Republic of Congo (DRC), Ethiopia, Gambia, Ghana and Mozambique, [22]. These countries were conveniently chosen based on the availability and willingness of the investigators to participate in this investigator-initiated study. In each country, clinics were selected based on the availability of anticoagulation services and laboratories for INR tests. Eligible patients received care from clinics chosen for at least three months and had at least four INR results.

### Sampling of participants and sample size

We applied a simple random sampling approach using random numbers generated by Excel/Stata to identify and select case records in clinics with more than or equal to 100 patients. All patients meeting the criteria were selected in clinics with fewer than 100 patients. We needed a pooled minimum sample size of 800 to estimate the suboptimal anticoagulation prevalence of 41% with a margin of error of 3.5% on a two-sided alpha level of 0.05 [18].

### Data collection procedures

Patient information was extracted from medical charts and electronic medical records. The information included demographic data (age, gender) indications, type and duration of VKA use and coexisting medical conditions. Other data were the international normalised ratio (INR) values, dates of INR testing and corresponding warfarin dosages for the 12-month duration during the study period.

### Assessment of anticoagulation control

We determined the level of anticoagulation using the Rosendaal and the Percent of INR in the therapeutic range methods [5, 8]. With the Rosendaal linear interpolation technique, we calculated the time-in-therapeutic range (TTR) in patients with at least four INR measurements whose consecutive INR testing time points were separated by 56 days or less [8]. We considered TTR less than 65% suboptimal anticoagulation control [23, 24]. The mean TTR for the cohort was the average of all TTRs, unadjusted for follow-up time included in the calculation. As there were centres where INR testing was done at intervals of more than 56 days, the anticoagulation control level was also calculated using the per cent of INR in the therapeutic range method by dividing the total number of INRs in the range for the cohort by the number of INRs [5, 25–27].. For both ways, the therapeutic INR range was 2.0–3.0 for venous thromboembolism and cardiac arrhythmia and 2.5 – 3.5 for mechanical prosthetic valves [7]. We used the results of the Rosendaal method for the bivariate and multivariate analysis of factors associated with anticoagulation control.

### Patient and public involvement

We did not directly involve patients in the study design, recruitment, and conduct.

### Statistical analysis

**Fig. 1:**
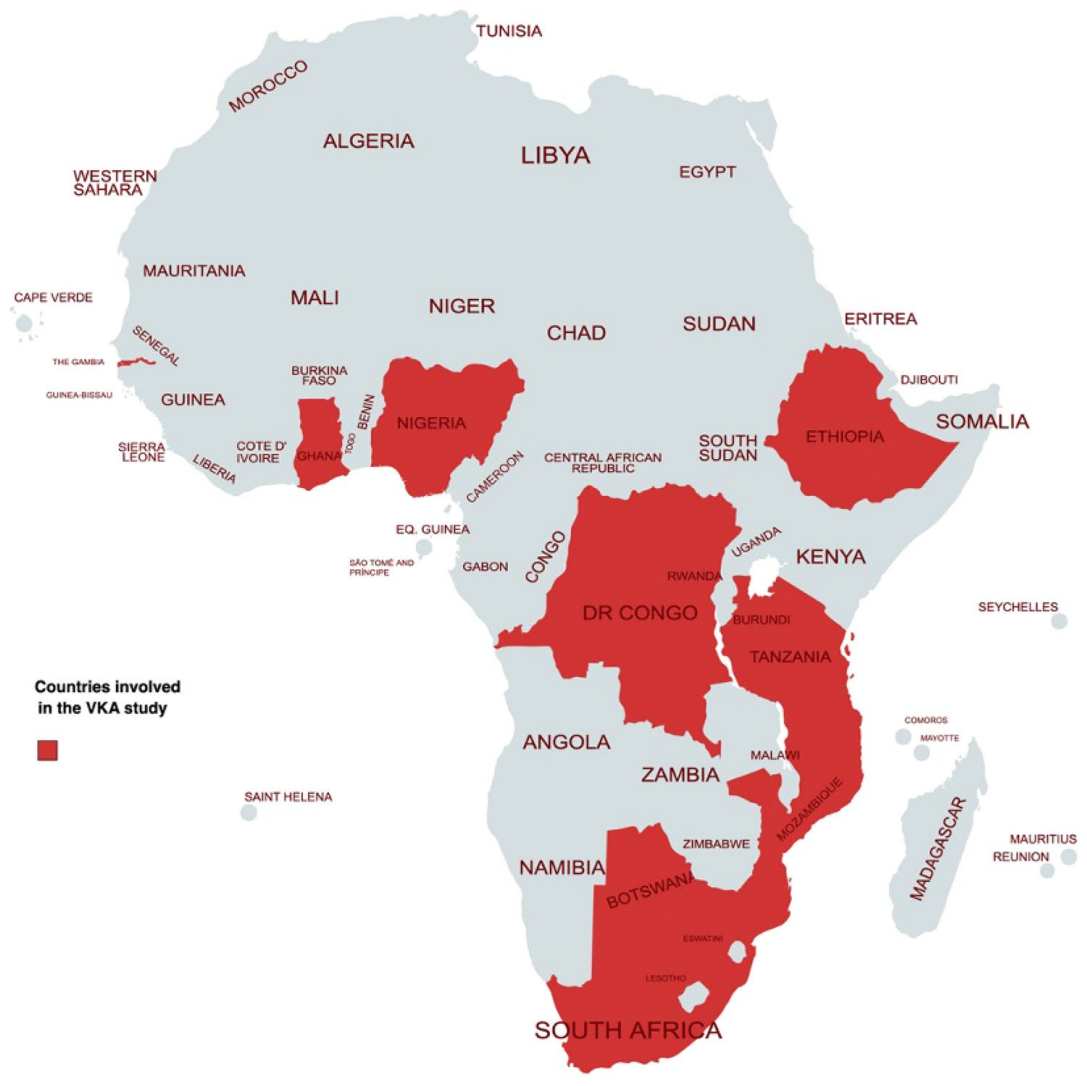
Countries involved in the VKA study

Data were entered into the Research Electronic Data Capture (REDCap) system hosted by the University of Botswana. We performed analyses using Stata V.14 (Stata Corp, College Station, Texas, USA). We used percentages to summarise categorical variables. Means and SD or medians and IQR were used to summarise continuous variables. We categorised deep vein thrombosis, pulmonary embolus, hepatic and splenic thrombosis, inferior vena cava thrombosis and cerebral venous thrombosis as venous thromboembolism (VTE). Intracardiac thrombus, Ischaemic stroke, dilated cardiomyopathy, antiphospholipid syndrome, peripheral arterial disease, thoracic aortic aneurysm, valvular heart disease, thrombophlebitis, carotid stent, femoral-saphenous junction blood stasis was classified as “other” indications for VKA. Countries with few participants, such as the Democratic Republic of Congo, Gambia, and Mozambique, were classified as “other”. Bivariate logistic regression explored factors associated with anticoagulation control. We further performed multivariable logistic regression models using clinically meaningful independent variables (indication for anticoagulation, medical service payment category, age groups and gender). Adjusted odds ratios (ORs), 95% confidence intervals (CIs), and *p*-values were documented. A 2-sided *p*-value < 0.05 was considered statistically significant (Fig. 1).

## Results

The study included 1011 patients whose mean age was 51 years (Table 1). Most participants (64%) were females, half were from Nigeria and South Africa, and half had an out-of-pocket payment for their medical. Warfarin was prescribed in 99.3% of the patients (n = 1004). The most common VKA indications for warfarin were venous thromboembolism, atrial fibrillation, and mechanical heart valves.

**Table 1:** Characteristics of patients anticoagulated in nine Sub-Saharan Africa, overall and by methods of anticoagulation control analysis (TTR), n = 1011

Characteristic	Eligible for TTR calculation n = 660	Ineligible for TTR calculation n = 351	All patients,
n = 1011			
Sex—n (%)			
Female	439(66.5)	210(59.8)	649(64.2)
Male	221(33.5)	141(40.2)	362(35.8)
Age, mean (SD), years	51.6(16.1)	50.3(16.1)	51.1(16.1)
Age group—n (%)			
< 40	170 (25.8)	99(28.2)	269(26.6)
40–49	131 (19.8)	74(21.1)	205(20.3)
50–59	140(21.2)	74(21.1)	214(21.2)
≥ 60	213(32.3)	103(29.3)	316(31.3)
Age unknown	6(0.9)	1(0.3)	7(0.7)
Anticoagulation			
Warfarin	657(99.5)	347(98.9)	1004(99.3)
Acenocoumarol	2(0.3)	4(1.1)	6 (0.6)
Phenprocoumon	1(0.2)	0	1(0.1)
Indication—n (%)			
Venous thromboembolism	223 (33.8)	76(21.7)	299(29.6)
Prosthetic valves	149(22.6)	121(34.5)	270(26.7)
Atrial fibrillation and flutter	198((30.0)	107(30.4)	305(30.1)
Other*	77(11.7)	41(11.7)	118(11.7)
Unknown	13(1.9)	6(1.7)	19(1.9)
Countries—n (%)			
Botswana	42(6.4)	58(16.6)	100(9.9)
Ethiopia	65(9.8)	31(8.8)	96(9.5)
Ghana	66(10.0)	34(9.7)	100(9.9)
Nigeria	134(20.3)	107 (30.5)	241(23.8)
South Africa	282(42.7)	31(8.8)	313(31.0)
Tanzania	37(5.6)	59(16.8)	96(9.5)
Other^‡^	34(5.2)	31(8.8)	65(6.4)
Medical service payment			
Out of pocket	334(50.8)	195(55.6)	529(52.4)
Universal coverage	240(36.2)	78(22.2)	318(31.4)
Insurance	86(13.0)	78(22.2)	164(16.2)

^*^Intracardiac thrombus (n = 25), Ischaemic stroke(n = 29), dilated cardiomyopathy(n = 15), pulmonary hypertension (n = 40); Anti-phospholipid Syndrome (n = 3); peripheral arterial disease(n = 1); thoracic aortic aneurysm(n = 1); valvular heart disease(n = 1), thrombophlebitis (n = 1), carotid stent(n = 1), scleroderma(n = 1)

^‡^Democratic Republic of Congo (n = 9), Gambia (n = 15), Mozambique (n = 41)

### Anticoagulation control

**Fig. 2:**
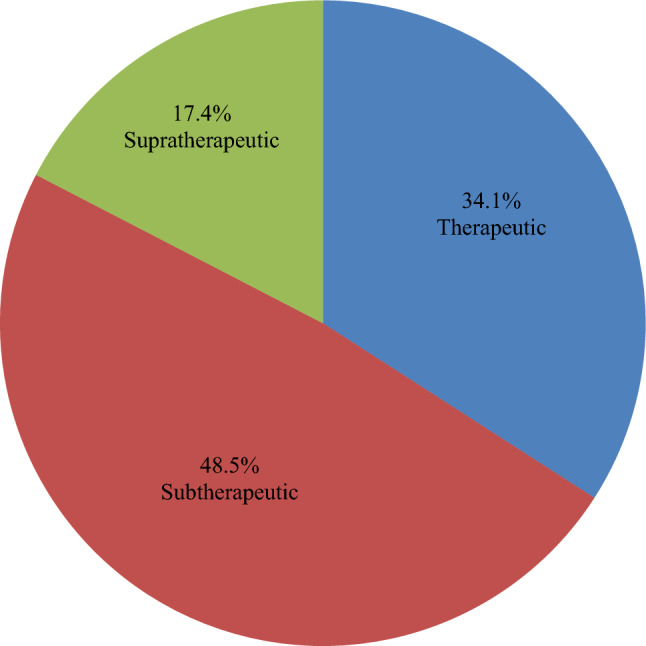
The proportion of INR results in sub-therapeutic, therapeutic, and supa-therapeutic (N = 6743)

We analysed 6743 INR tests from all the 1011 participants, with each patient having a median (IQR) of 6(4,9) tests. Of these tests, 48.5% were subtherapeutic, 34.1% therapeutic, and 17.4% were supratherapeutic relative to disease-specific reference ranges (Fig. 2).

### The time in therapeutic range (TTR)

Of the 1011 participants, 660(65.3%) were eligible for the TTR assessment. The median (IQR) observation period was 118 (84,174) days. The median (IQR) TTR was 35.7(15.9, 57.3) %, ranging from 0% in Nigeria to 44.6% in Ghana (Table 2). The proportion of patients with optimal anticoagulation was 19.2%, varying from 2.7% in Tanzania to 23.1% in Ethiopia.

**Table 2:** The Median TTR and the proportion of patients with ≥ 65% by countries, VKA indications and age group of participants (N = 660)

Characteristic	Median TTR(IQR), %	Optimal anticoagulation control
Overall	35.8(15.9, 57.2)	127(19.2)
VKA indications—n (%)		
Venous thromboembolism	38.2(18.2, 59.4)	47/223(21.1)
Prosthetic valves	27.4(13.7, 48.0)	23/149 (15,4)
Atrial fibrillation and flutter	43.7(22.4,64.2)	47/198(23.7)
Others*	27.7(0, 54.9)	9/77 (11.7)
Unknown	11.1(0,31.4)	1/13(7.7)
Age group—n (%)		
< 40	28.2(5.0, 55.4)	30/170(17.6)
40–49	31.3(5.2, 57.0)	23/131(17.6)
50–59	38.2(19.5, 52.6)	19/140(13.6)
≥ 60	41.8(22.6,65.2)	54/213(25.4)
Age unknown	25.5(18.3,36.9)	1/6(16.7)
Countries—n (%)		
Botswana	21.5(9.9, 46.7)	6/42(14.3)
Other^‡^	40.9(23.9,51.6)	5/34(14.7))
Ethiopia	39.7(21.9,64.2)	15/65(23.1)
Ghana	44.6(32.3, 62.8)	14/66(21.2)
Nigeria	0(0, 45.3)	22/134(16.4)
South Africa	40.6(23.7, 61.6)	64/282(22.7)
Tanzania	20.9(9.7, 41.4)	1/37(2.7)
Mode of payment—n (%)		
Out of pocket	32.7(5.9,54.4)	57/335 (17,0)
Universal coverage	40.4(20.5,64.1)	59/239 (24,7)
Private insurance	35.2(20.8, 55.0)	11/86(12.8)

*Intracardiac thrombus (n = 20), Ischaemic stroke(n = 19), dilated cardiomyopathy(n = 14), pulmonary hypertension (n = 15) Anti-phospholipid Syndrome (n = 2), peripheral arterial disease(n = 2), thoracic aortic aneurysm (n = 1),, valvular heart disease(n = 1), thrombophlebitis (n = 1), carotid stent thrombosis(n = 1), scleroderma(n = 1),

^‡^Democratic Republic of Congo(n = 5), Gambia, (n = 2) Mozambique (n = 24)

### Factors associated with anticoagulation control

Countries with universal health coverage had higher odds of optimal anticoagulation control (adjusted odds ratio (aOR) 1.79, 95% confidence interval [CI], 1.15– 2.81, p = 0.01) (Table 3).

**Table 3:** Multivariable Logistic Regression model for association with adequate anticoagulation control (N = 660)

	N (%)^¥^	Crude OR	p-value	Adjusted OR	p-value
Sex					
Female	85/354(19.4)	1		1	
Male	42/179(19.0)	0.98(0.65–1.47)	0.91	1.03(0.67–1.59)	0.89
Age group—n (%)					
< 40	30/170(17.6)	1		1	
40–49	23/131(17.6)	0.98(0.55–1.81)	0.98	1.01(0.55–1.86)	0.97
50–59	19/140(/13.6)	0.73 (0.39–1.37)	0.33	0.65(0.34–1.24)	0.19
≥ 60	54/213(25.4)	1.59(0.96– 2.62)	0.07	1.27 (0.75–2.14)	0.36
Age unknown	1/6(16.7)	0.95(0.11–8.28)	0.95	0.97(0.05 –6,41)	0.98
VKA indications					
Venous thromboembolism	47/223(21.1)	1		1	
Prosthetic valves	23/249 (15.4)	0.68(0.40–1.18)	0.17	0.56(0.31–1.00)	0.05
Atrial fibrillation and flutter	47/198(23.7)	1.17(0.74–1.85)	0.51	1.14 (0.71–1.83)	0.60
Other*	9/77(11.7)	0.50 (0.23–1.07)	0.07	0.52(0.24–1.14)	0.10
Unknown	1(/13(7.7)	0.31(0.04–2.46)	0.27	0.33 (0.02 –1.80)	0.30
Medical service payment					
Out of pocket	57/334(17.1)	1		1	
Universal coverage	59/240(24.6)	1.53(1.02–2.30)	0.04	1.79 (1.15–2.81)	0.01
Insurance	11/86(12.8)	0.70 (0.35–1.40)	0.31	0.75 (0.36–1.48)	0.42

^*^Intracardiac thrombus (n = 20), Ischaemic stroke(n = 19), dilated cardiomyopathy(n = 14), pulmonary hypertension (n = 15) Anti-phospholipid Syndrome (n = 2), peripheral arterial disease(n = 2), thoracic aortic aneurysm (n = 1),, valvular heart disease(n = 1), thrombophlebitis (n = 1), carotid stent thrombosis(n = 1), scleroderma(n = 1),

## Discussion

We sought to determine the anticoagulation control among patients on VKA in SSA. Data from our study indicate that the proportions of INR values in the therapeutic range, the median TTR and the proportion of patients with TTR ≥ 65% are all low. In all nine countries, the median TTR values were below 65%, ranging from 0 to 44.6%. In addition, we consistently observed poor anticoagulation control in patients with all VKA indications (mechanical heart valves, atrial fibrillation, and venous thromboembolism). For those with suboptimal control, under-anticoagulation is more frequent than over-anticoagulation. Universal health coverage was more likely associated with optimal anticoagulation control. Our analysis also showed a lower frequency of INR measurements among the study participants.

The high burden of suboptimal anticoagulation control in the current study is consistent with the findings in similar studies in SSA, where the TTR values ranged from 13.7% to 47% and the proportion of patients with TTR ≥ 65% as low as 15% [16, 28]. Our findings are consistent with the global GARFIELD study, which also reported a low proportion of patients with TTR ≥ 65%, varying from 16.7% in Asia to 49.4% in Europe [13]. Therefore, real-world data, including ours, indicate that optimal anticoagulation is often not achieved in routine clinical practice. This apparent suboptimal anticoagulation in VKA-treated individuals is concerning, given the increased mortality risk of as much as fourfold when TTR falls below 30% [15]. Our findings strengthen the argument for improving anticoagulation quality, particularly in SSA, where VKAs are the primary anticoagulants [13]. Evidence suggests that VKA at stable optimal anticoagulation has comparable efficacy and safety to NOACs [19–21].

The relatively high proportion of sub-therapeutic INRs is consistent with a previous meta-analysis and meta-regression conclusion, which reported predominantly under-anticoagulated in patients on VKA anticoagulation [14]. Although most studies typically report 25–36% of INRs below the range, the proportion of sub-therapeutic INRs in our cohorts was almost 50% [13, 14, 29–32]. Our findings not only depict suboptimal anticoagulation control but also that when patients were out of range, they were more likely to be sub-therapeutic and at an increased risk of thrombosis than supratherapeutic with an increased risk of bleeding. This is a concern as subtherapeutic anticoagulation may cause up to a 16-fold increase in the rate of thromboembolism [33]. Though the reasons are unclear, subtherapeutic anticoagulation is likely related to a lack of dedicated anticoagulation clinics and less stringent monitoring, VKA non-adherence, dosage interruption for several reasons, a recent dose reduction in response to a previously recorded high INR value and clinicians' using low dosage due to fears of inducing a major haemorrhage [33]. Regardless of the reason, our results call for efforts to reduce or eliminate sub-therapeutic anticoagulation to improve patient outcomes.

Better anticoagulation control has been attained in clinical trials performed in the SSA [2]. In the Investigation of Rheumatic AF Treatment Using VKA, Rivaroxaban or Aspirin Studies (INVICTUS) trial that involved several African countries, 56.1% to 65.3% of INR tests were in the therapeutic range during the four years of follow-up, rising from 33.2% before the trial. The INVICTUS study reported a lower rate of a composite of stroke, systemic embolism or myocardial infarction, or death in patients with rheumatic heart disease–associated atrial fibrillation on warfarin than rivaroxaban therapy. The improved quality of anticoagulation is a significant determinant of thrombotic and bleeding events in patients receiving a vitamin K antagonist and likely influenced outcomes in the INVICTUS trial. Although these results show that optimal quality of anticoagulation can be achieved in resource-limited countries, the trial's rigorous follow-up and strict inclusion criteria make it difficult to extrapolate the results to real-life VKA-treated patients [2]. Due to frequent anticoagulation monitoring, patients in the VKA arm had frequent physician interactions, which could have resulted in better clinical outcomes. Our study analysed anticoagulation data routinely collected from different levels of healthcare and likely reflects the accurate picture of the quality of anticoagulation in non-trial clinical sub-Saharan African settings. Putting these results into perspective, the benefits seen in the INVICTUS trial are difficult to replicate in sub-Saharan Africa, where time, distance, economic or other access-to-care issues impede access to anticoagulation care.

Our analyses noted an association between optimal quality of anticoagulation and universal health coverage. This finding is consistent with previous evidence linking out-of-pocket expenses for VKA services and suboptimal quality of the anticoagulation [13, 16, 18]. Owing to the underfunding of the health systems in Africa, patients travel a long distance to access the few unreliably running centralised VKA services [17].

### Limitations

The limitations of this study are those typical of a retrospective design. Although the analysis of variables in medical charts and electronic records was limited by missed information, the extracted information provided an accurate picture of the quality of anticoagulation in daily clinical practice. The convenient inclusion of sites and investigators may have potentially selected more motivated investigators and participants, with a consequential overestimation of the anticoagulation control. The distribution of the clinics in selected countries may not have yielded a representative sample as they were predominantly in urban settings. However, most anticoagulation services in SSA are often centralised, caring for urban and rural patients who often travel long distances to access these services[17]. The small number of participants in some countries and clinics may also have affected the representativeness of our sample and the generalisation of the findings in such settings. However, the quality of anticoagulation management in our analysis is consistent with that reported in other studies in SSA. Therefore, our results can be extrapolated to most SSA settings. Although data entries from different sites may have been prone to errors, site investigators were responsible for supervising and ensuring correct data entry. In addition, the REDCap dataset included customised validation rules to ensure that valid data are entered.

## Conclusion

In conclusion, patients on VKAs for different therapeutic indications in SSA are frequently outside the therapeutic INR range and tend to be sub-therapeutic rather than over-anticoagulated. Anticoagulation care in settings without universal health is associated with suboptimal anticoagulation control. The evidence calls for efforts to improve the region’s anticoagulation control among patients in sub-Saharan Africa, including providing VKA services free of charge.
