# Correction to: Anticoagulation control among patients on vitamin K antagonists in nine countries in SubSaharan Africa

**DOI:** 10.1007/s11239-024-03037-3

**Published:** 2024-09-30

**Authors:** Julius Chacha Mwita, Joel Msafri Francis, Chriselda Pillay, Okechukwu S. Ogah, Yadeta Dejuma Goshu, Francis Agyekum, John Mukuka Musonda, Maduka Chiedozie James, Endale Tefera, Tsie Kabo, Irene Keolebile Ditlhabolo, Kagiso Ndlovu, Ayoola Yekeen Ayodele, Wigilya P. Mikomangwa, Pilly Chillo, Albertino Damasceno, Aba Ankomaba Folson, Anthony Oyekunle, Erius Tebuka, Fredrick Kalokola, Karen Forrest, Helena Dunn, Kamilu Karaye, Fina Lubaki Jean-Pierre, Chala Fekadu Oljira, Tamrat Assefa, Tolulope Shogade Taiwo, Chibuike E.Nwafor, Olufemi Omole, Raphael Anakwue, Karen Cohen

**Affiliations:** 1 Department of Internal Medicine, University of Botswana and Princess Marina Hospital, Gaborone, Botswana; 2 Department of Family Medicine and Primary Care School of Clinical Medicine, Faculty of Health Sciences, University of the Witwatersrand, Johannesburg, South Africa; 3 Division of Clinical Pharmacology, Department of Medicine, University of Cape Town, Cape Town, South Africa; 4 Cardiology Unit, Department of Medicine, University of Ibadan/University College Hospital, Ibadan, Oyo State Nigeria; 5 Department of Internal Medicine, College of Health Sciences, Addis Ababa University, Addis Ababa, Ethiopia; 6 Department of Medicine, College of Health Sciences, Korlebu Teaching Hospital, University of Ghana, Ghana, Ethiopia; 7 Federal Medical Centre (FMC), Umuahia, Abia State Nigeria; 8 Department of Paediatrics and Adolescent Health, University of Botswana and Princess Marina Hospital, Gaborone, Botswana; 9 Department of Computer Science, University of Botswana, Gaborone, Botswana; 10 Cardiology Unit, Department of Medicine, Federal Teaching Hospital, Gombe, Gombe State Nigeria; 11 Department of Clinical Pharmacy and Pharmacology, Muhimbili University of Health and Allied Sciences, Dar es Salaam, Tanzania; 12 Department of Internal Medicine, Muhimbili University of Health and Allied Science, Dar es Salaam, Tanzania; 13 Faculty of Medicine, University Eduardo Mondlane, Maputo, Mozambique; 14 University of Health and Allied Sciences, Accra, Ghana; 15 Department of Internal Medicine, Bugando Medical Centre, Mwanza, Tanzania; 16 MRC Unit The Gambia at LSHTM, Fajara, The Gambia; 17 Department of Medicine, Bayero University & Aminu Kano Teaching Hospital, Kano, Nigeria; 18 Department of Family Medicine and Primary Care, The Protestant University of Congo, Kinshasa, Democratic Republic of the Congo; 19 Department of Pharmacology and Clinical Pharmacy, College of Health Sciences, Addis Ababa University, Addis Ababa, Ethiopia; 20 University of Uyo Teaching Hospital, Uyo, Nigeria; 21 The University of Port Harcourt Teaching Hospital, Port Harcourt, Nigeria; 22 Departments of Medicine, Pharmacology/Therapeutics, The University of Nigeria Teaching Hospital, Enugu, Nigeria

**Journal of Thrombosis and Thrombolysis (2024) 57:613–621**

10.1007/s11239-023-02928-1.

In this article, the affiliation details for Author John Mukuka Musonda were incorrectly given as ‘Division of Clinical Pharmacology, Department of Medicine, University of Cape Town, Cape Town, South Africa’ but should have been ‘Department of Family Medicine and Primary Care School of Clinical Medicine, Faculty of Health Sciences, University of the Witwatersrand, Johannesburg, South Africa’.

And the author’s names Wigilya P. Mikomangwa and Tamrat Assefa was incorrectly written as Wigilya P.Mkomanga and Tamirat Assefa Tadesse.

The Original article has been corrected.

